# Criticality of Timing for Anti-HIV Therapy Initiation

**DOI:** 10.1371/journal.pone.0015294

**Published:** 2010-12-23

**Authors:** Filippo Castiglione, Paola Paci

**Affiliations:** 1 Institute for Computing Applications “Mauro Picone”, National Research Council of Italy, Rome, Italy; 2 Biomedical University Campus, Rome, Italy; Dana-Farber Cancer Institute, United States of America

## Abstract

The time of initiation of antiretroviral therapy in HIV-1 infected patients has a determinant effect on the viral dynamics. The question is, how far can the therapy be delayed? Is sooner always better? We resort to clinical data and to microsimulations to forecast the dynamics of the viral load at therapy interruption after prolonged antiretroviral treatment. A computational model previously evaluated, produces results that are statistically adherent to clinical data. In addition, it allows a finer grain analysis of the impact of the therapy initiation point to the disease course. We find a swift increase of the viral density as a function of the time of initiation of the therapy measured when the therapy is stopped. In particular there is a critical time delay with respect to the infection instant beyond which the therapy does not affect the viral rebound. Initiation of the treatment is beneficial because it can down-regulate the immune activation, hence limiting viral replication and spread.

## Introduction

According to an estimation, the AIDS pandemic has killed about 2.1 million people, including 330,000 children and about 33.2 million people lived with the disease worldwide [1]. Notwithstanding the exceptional scientific effort to find a ultimate cure to this immune deficiency disease, there is no definite treatment to eradicate the virus from infected people to date. What has been achieved up to date is a prolonged life expectancy by using antiretroviral cocktails (highly active antiretroviral therapy, shortly HAART) that, unfortunately, have to be administrated throughout the life of the patients. Since the uptake of these therapeutic agents in forms of pills is a significant burden both in terms of patients commitment and health care costs, there is a huge interest in understanding when is the golden moment to initiate the therapy. The question is whether it is possible to delay the initiation of the therapy while the potency of the immune response remains unaffected or, conversely, *is sooner always better for anti retro-viral therapy*? This issue still remains a challenge although recent studies agree that an early initiation of the therapy can influence positively the course of the disease [2]. Hereafter we show that this is indeed our same conclusion by comparing clinical data with computer simulations. Moreover, while current antiretroviral therapy is efficacious only if applied with continuity, it is of utter interest to study its effect on the dynamics of the virus; by simulating on a computer the infection, before, during and after the therapy, we can get insights otherwise difficult or even impossible to be deduced *in vivo*.

The early immune response to HIV-1 infection looks likely to be an important factor in determining the clinical course of disease. If we watch the first weeks following HIV-1 invasion we find that they are extremely dynamic. We learn that they are associated with a hasty damage to the generative immune cell micro-environment and with an immune response that is only partially able to control the virus. Once inside the host body, the virus first replicates locally in the mucosa and then is transported to draining lymph nodes. There, further viral amplification occurs. In this initial phase the plasma viremia increases exponentially and reaches a peak. It terminates when systemic viral dissemination begins [2]. This phase is associated with significant depletion of mucosal CD4
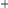
T cells. At around the peak time, the disease may become symptomatic and, more importantly, reservoirs of latent virus are established [3]. Following this initial “acute phase”, lasting approximatively 100 days after the infection, the beginning of the chronic phase is characterized by a plateau in the plasma viral load.

The acute phase is generally divided in the *very-early* and *early* phases. The *very-early* is asymptomatic, whereas the *early* is characterized by the appearance of clinical symptoms [4]. Unsurprisingly, quite a number of researchers has pointed to the early infection period around the peaking of viremia, prior to massive CD4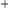 T cell destruction and the establishment of viral reservoirs, as a narrow but crucial period in which the antiretroviral therapy can secure a certain advantage on the virus, controlling its replication, preventing extensive CD4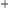 T cell depletion from occurring, and curbing generalized immune activation [2], [3]. Hence, there is a “window of opportunity” to identify. Animal models of AIDS would provide a workbench to study this issue. However, the only animals susceptible to experimental HIV-1 (or HIV-2) infection are the chimpanzee, macaques, gibbon apes and rabbit but unfortunately for us (not them), AIDS-like disease has not yet been reported in these species. For what concerns the HIV-infected SCID-hu mice, it is not yet clear how suitable will it be as a model for AIDS. Another possibility is to study one of the several subfamilies of naturally occurring retroviruses that cause immune suppression. For example the feline immunodeficiency virus (FIV) infecting cats and the lentivirus simian immunodeficiency virus (SIV) infecting macaques appear to bear the closest similarity in their pathogenesis to HIV infection and AIDS [5]. In either case, leaving out ethical arguments, an important drawback of animal experimentation is its cost, both in terms of time and money. Here is where computational models come handy. They try to resemble, in the most realistic yet parsimonious way, the events taking place during the infection of a virus.

In the present work we check out whether the effect of a timely HAART initiation can prevent the virus to damage the host defenses and to lodge in cellular and anatomical reservoirs thus assuring the patient a nearly regular life. We do this by analyzing clinical data and simulations performed with a model of HIV-1 infection that has previously shown to be a valuable tool for the study of the AIDS progression and treatment [6], [7]. The computer simulation allows a finer grain study than clinical data does.

## Results and Discussion

For the current study we analyze virological data from HIV patients treated during the *very-early*, *early* and *late* phase of infection and compare them with computer simulations. Clinical data were collected at the National Institute for Infectious Disease “L. Spallanzani”. We classify a total of 54 patients in three groups according to the time they initiated the therapy. Eleven patients initiated HAART within 20 days from diagnosis, during the *very-early* phase of infection, before symptoms begin. Twenty-two patients underwent HAART during the *early* phase of HIV-1 infection. Twenty-one patients started HAART during the chronic phase of infection thus in the *late* phase. All patients underwent a therapy cycle for about a year.

**Figure 1:**
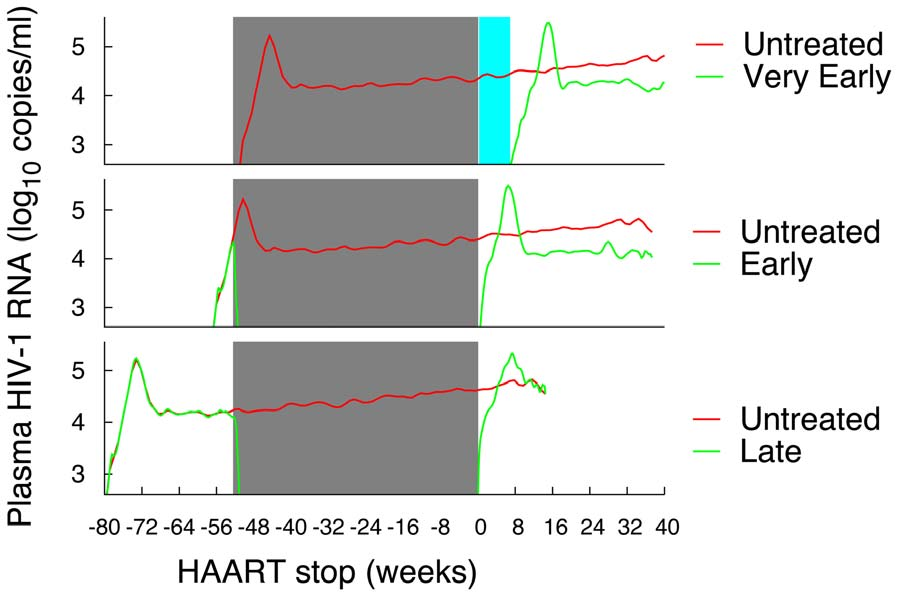
Simulations of treated and untreated case. Comparing simulations of the treated and untreated cases. All simulations share the same parameter settings with the only difference that therapy starts at day 0 in the *very-early*, at day 40 in the *early* and at day 200 in the *late* settings. The duration of simulated therapy is one year.

Statistical analysis of the clinical data of the three groups reveals that there is no difference in viral rebound between *early* and *late* patients (P
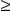
0.05, Mann-Whitney U two-tailed test) whereas we find a difference between *very-early* and *late* (P
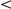
0.05, Mann-Whitney U two-tailed test). Simulations are in line with this finding. This is shown in Figure 1 that compares a single simulation of the untreated case with respectively a *very-early*, *early* and *late* settings. As a first observation, in agreement with clinical data, we note that as soon as the therapy stops the simulated viremia readily increases and peaks very much like in the acute phase of the untreated case. Even more interesting is to notice the swift viral rebound at therapy discontinuation in the *early* and *late* cases. Instead, there is quite a long delay (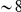 weeks) in the *very-early* case (cyan area in Figure 1). The gist of the message is that a delay in the initiation of therapy may give the virus the chance to damage to the generative immune cell micro-environment and establish latent virus reservoirs [2], [3]. Therefore, despite the fact that the virus emerges again at therapy discontinuation regardless of when the therapy started, its early control influences positively the clinical course of the disease.

Since there is no statistical difference between the *early* and *late* groups, from now on, we concentrate on the analysis of the gap between the *very-early* and *late* groups only. To this purpose we can safely focus on the time point of 4 weeks after therapy discontinuation, hereafter indicated by 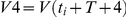, where 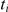 is the time of initiation of the therapy in weeks and 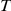 is its duration.

**Figure 2:**
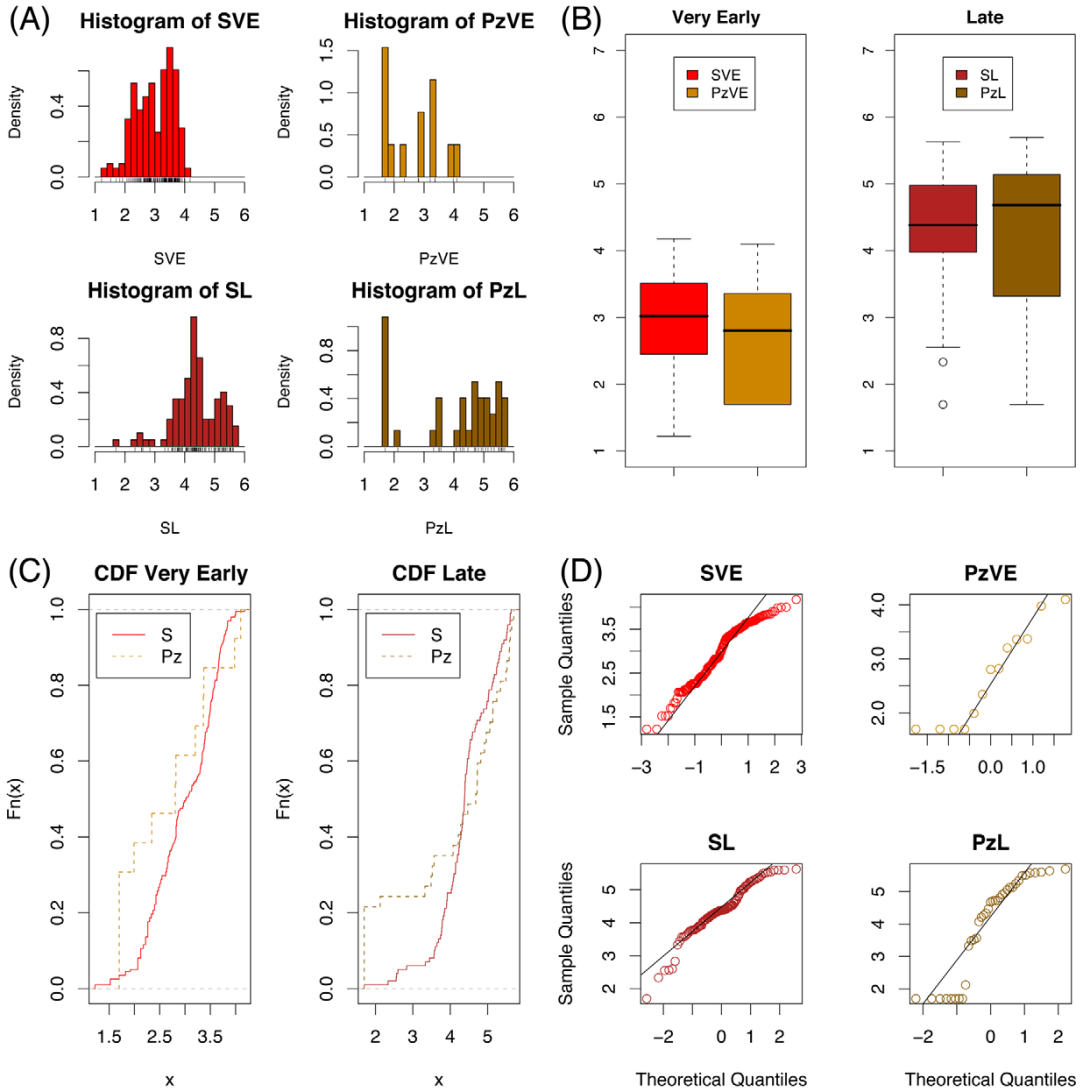
Clinical and simulated data. Statistical summary of clinical and simulated 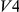 for both *very-early* and *late* settings. Panel A shows how the data is distributed. In panel B the “whiskers” indicate the lower, median and upper quartile while largest and smaller values are shown by dashed lines. Outliers are shown as small circles. Panel C shows the cumulative distribution functions for the two groups. Panel D reports the normal probability plots (or quantile-quantile plots) for comparison to normal distributions. Legend: SVE  =  simulated very early, PzVE  =  patients very early; SL  =  simulated late, PzL  =  patients late.

Before using the simulation to predict the relationship between the initiation of the therapy and the viremia at its discontinuation, we have to assess its adherence to clinical data. To this purpose we carry out a statistical test (*chi-square goodness of fit test* [23]) and find that with a significance level of more than 90% the simulations are adherent to clinical data. Furthermore, in Figure 2, panels A, B and C, graphically compare the clinical and simulated 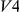 for both *very-early* and *late* settings. In particular, in panel B the “whiskers” indicate that the median of simulated and real values are pretty close, whereas panel C shows that the cumulative distribution functions for the two groups overlap quite well.

The use of the statistical parametric test to evaluate the relationship between the initiation of the therapy and the virological rebound 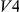 urges to check whether the distribution of the simulated 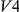 in *very-early* and *late*, can be fitted by a normal distribution. To this purpose panel D of Figure 2 shows the quantile-quantile plots of the four data sets; data aligned on a line demonstrates adherence to the normal distribution. Besides, we perform a *chi-square goodness of fit test* [23] and find that with a 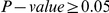 both simulated *very-early* and *late* 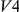 data are normally distributed. Finally we use a *chi-square test for independence* [23] to demonstrate that within a reasonable significance level the viral rebound at discontinuation 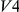 strongly correlates with the therapy initiation instant 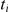 (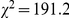).

All the above statistical analysis allow to use the simulation as a surrogate of reality and therefore to investigate whether there is a significant association between 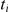 and the viral rebound. The sample data is summarized in the contingency Table 1. Performing the test we find 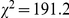. The high value of the statistics leads to the conclusion that, within a reasonable significance level, the viral rebound at discontinuation strongly correlates with the therapy initiation instant.

**Table 1:** Contingency table.

Viral	Late	Very early	Total
rebound	(pz)	(pz)	(pz)
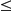 3	6	98	**104**
3–4	19	98	**117**
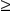 4	74	2	**76**
**Total**	**99**	**198**	**297**

Contingency table for simulated data where the expected frequency count for each cell of the table is at least 5. The rows represent the average viremia after 4 weeks from treatment interruption in logarithmic scale. The columns represent the two groups in which HAART starts during chronic phase (*late*) or within the first 20 days (*very-early*). The null hypothesis states that knowing the start timing does not help to predict 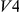. That is, the events are independent. Support for the alternative hypothesis suggests that the two events are related. The value found 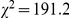 rejects the null hypothesis.

**Figure 3:**
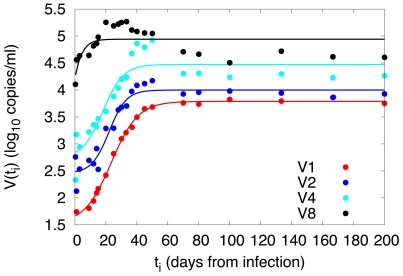
Virological rebound after stopping HAART. Virological rebound at 1, 2, 4 and 8 weeks after therapy interruption for different starting time points of HAART (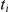). Dots represent the results of thousands simulations. The fitting lines are given by the Richards' curve in equation 1. Standard deviation is about 0.1 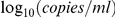 for all points.

We aim at providing a more precise estimate of the time “limit” beyond which the benefit of an early initiation of therapy vanishes. The results are shown in Figure 3. The virological rebound at one, two, four and eight weeks after therapy interruption (called respectively 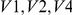 and 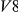) as a function of 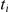 are presented. The points in Figure 3 are fitted pretty well a generalized (Richards', [24]) logistic function describing the growth of viremia as a function of 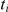,
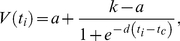
(1)where the parameter 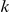 is the carrying capacity or the upper asymptote, 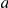 is the lower asymptote, 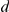 is the growth rate, and 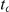 is the time of maximum growth.

We observe that there are two regimes, one for 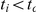 days and one for 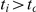 corresponding to what clinicians call respectively *best controllers* (with undetectable HIV RNA levels) and *rebounders* (whose HIV viremia load returns, approximately, to the pre-HAART level). Therefore 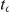 can be seen as a critical time point beyond which the benefit of an early initiation of therapy vanishes.

By moving the time of the measurements beyond one week after therapy interruption (*i.e.,* 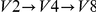), the resulting fit corresponds to a greater 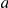, a smaller 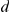 and a lower 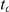. Note in particular that the limit
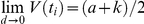
may lead to the deceiving conclusion that there is no window of opportunity because the viral rebound is independent from 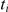. However, from the test performed on V4 we know that this is not the case. Along the same line, we note that 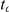 depends on *when* the viremia is measured: the estimated 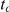 is equal to about 24, 22, 18 and 9 days respectively for 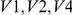 and 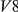.

### Conclusions

HAART is costly, it is demanding for both patient and health care provider, and it brings quite frequently to adverse effects. The clinical benefit of treatment must therefore be weighed against the burden imposed by therapy and its side-effects. In the present study, we resort to a computer model to forecast the dynamics of the plasma viral load after prolonged treatment interruption and compare the results with clinical data. Our conclusion, in line with literature data, is that very early initiation of the treatment is beneficial because it can down-regulate the immune activation, hence limiting viral replication and spread. Interestingly, this view is supported by the observation that HIV triggers the immune activation directly (*e.g.,* HIV gene products can induce the activation of lymphocytes and macrophages as well as the production of pro-inflammatory cytokines and chemokines [3]) or indirectly (*e.g.,* sustained antigen-mediated immune activation occurs in HIV-1-infected patients due also to other viruses like the cytomegalovirus or the Epstein-Barr virus [3]). In both case, the result is a high level of pro-inflammatory cytokines, such as tumor necrosis factor alpha, interleukin 6 and interleukin 1 beta, right from the early stages of HIV-1 infection [3].

Simulations and statistical analysis allow to dig into clinical data to provide a clear cut evidence of the impact of the therapy initiation point to the disease course.

## Methods

### The computational model

The microscopic simulation model we employ has a long history. It is perhaps one of the oldest computational models of the immune system, dating back to the early nineties [8]. The current version [9] we use derives from that early model and has been specialized to simulate the HIV-1 infection some time ago [6]. In [10] we have described how the HIV evades immune surveillance by mutation; in [7] we have shown that early application of HAART is more likely to be beneficial than the deferred one. We have also used this model coupled with genetic algorithms to determine the best therapy interruption protocol [11]. This same model has also been used to describe other disease courses: in [12] we have shown that the Epstein-Barr virus cannot be cleared by the immune system because it exploits reservoirs; in [13], [14] we have modeled the effects of a cancer immunoprevention vaccine.

The utility of this model is that with the greatest fidelity possible, yet avoiding to end up with an unmanageable model, it represents the basic facts of the immune action solicited by an infectious agent. For this reason, every lesson learned in previous studies including parameter tunings, has become an integral part of the model itself. Mechanisms like hypermutation, affinity maturation and so on, have already been investigated and their implementations have now “crystallized” into the core model.

**Figure 4:**
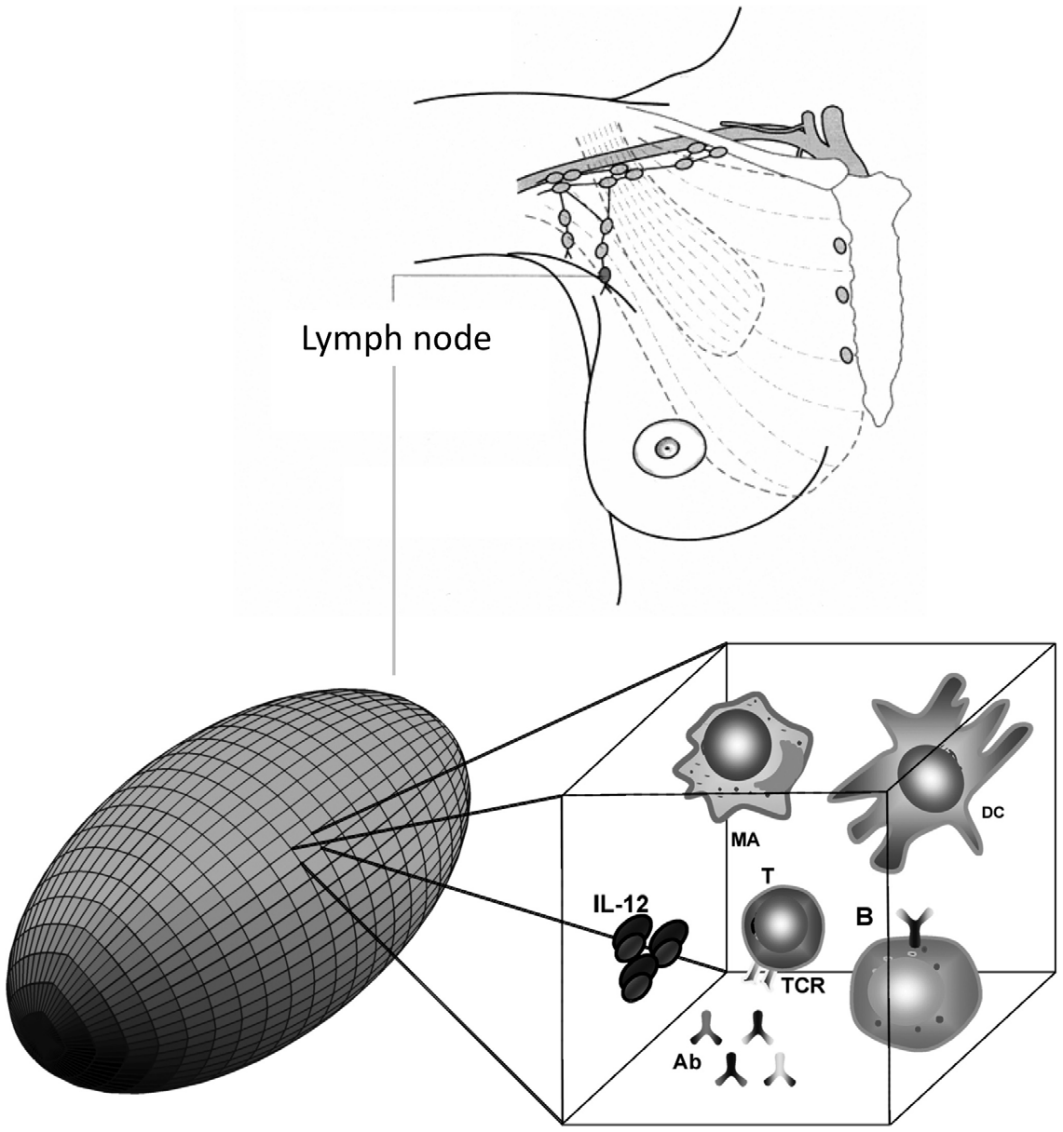
Simulation space. The ellipsoid lattice resemble the typical shape of a lymph node. Entities interact within voxels. Typical simulations consist of 5 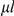 of volume discretized on 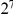 voxels and populated by about 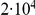 cells.

The simulator represents immunological cells and molecules as discrete entities residing and interacting on a three-dimensional regular lattice. Each lattice point represents a unit of volume, that is, a *voxel*. The simulated total volume corresponds to a fraction of a lymph node (see Figure 4). As in cellular automata models, time is discrete and the state of a lattice point at time 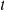 is a function of a finite number of variables, namely the biological entities residing on that voxel. Agents are cells and molecules with specific characteristics (*i.e.,* molecular receptors, half life, etc.). At any time each agent can be in one of a set of possible states (naïve, active, resting, duplicating, etc.). Probabilistic rules define the biological processes by modifying the state of the entities. We can write 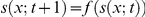 where 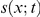 is the state of the voxel 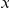 given as the union of the microstates of all entities in 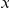, that is, 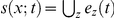, with the index 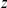 running on all entity types and 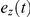 representing the value at time 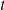 of the attributes of entity type 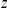. The function 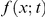 embeds many biological processes like cell differentiation, cell interaction and movement. Another characteristic of this model is that all molecules and cells' *binding sites* (*e.g.*, cell receptors) are modeled as binary strings of finite length. Models of this kind are called *binary-string models* [15]. Each interaction requires cell entities to be in a specific state. Once this condition is fulfilled, the interaction probability is computed as a function of the distance between receptors. Small molecules (*i.e.*, molecules with small molecular weight like *interleukins* or *chemokines* that are carriers of physiological signals, are represented as concentrations and thus their dynamics can be described by partial differential equations of the parabolic type
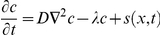
where 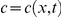 is the concentration of chemokines, 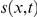 is the source term (*e.g.*, activated macrophages), 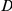 is the diffusion coefficient and 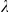 is the half-life [16], [17]. Instead, the differences in the mobility of the cells is taken into account by implementing a biased random walk that considers experimentally estimated diffusion coefficients [18], [19]. These are the main features. The interested reader may look at more comprehensive publications for further details [6], [7], [20] and in the supplementary materials.

### Clinical studies

We analyze the results of clinical studies performed at the Clinical Department of the National Institute for Infectious Disease “L. Spallanzani” in Rome.

A first group of eleven patients (9 male and 2 female) were diagnosed HIV-1 positive between year 1998 and 2006. All patients initiated HAART within 18 days from diagnosis, during the very early phase of infection. The very early phase was defined as having a negative or indeterminate western blot for HIV-1 antibodies in combination with a positive test for either p24 antigen or a detectable HIV-1 RNA concentration. Those patients were treated with zidovudine/lamivudine (CBV) in combination with either the reverse transcriptase inhibitor efavirenz (EFZ) or one protease inhibitor lopinavir/ritonavir (KAL) or indinavir (IDV). Because of anaemia and neutropenia were diagnosed, in two cases CBV has been substituted with lamivudine (3TC) and staduvine (D4T).

The second group consists of twenty-one patients (12 male, 9 female) enrolled in the program between year 1990 and 2004. They started HAART during the chronic phase of infection defined as suggested by the guidelines [21].

The third group is made up by twenty-two patients (21 male and 1 female) enrolled in the program between year 1998 and 2005. Patients in this group underwent HAART during the early phase of HIV-1 infection. Early patients were defined as having documented seronegative HIV-1 antibody test within the previous 6 months; acute symptomatic seroconversion illness; evolving HIV-specific antibody response by ELISA; positive HIV-DNA PCR in PBMC. Those patients were treated with three different drugs (in the majority of cases zidovudine (AZT) plus 3TC plus a protease inhibitor. Further details can be found in table 1 of [22].

All patients underwent a therapy cycle for about a year.

All clinical investigation have been conducted according to the principles expressed in the Declaration of Helsinki. The Ethical Committee of the “L. Spallanzani” Institute approved the study and the patients gave a written informed consent.

### Plasma HIV-1 determination

Plasma HIV-1 RNA levels were determined by a second-generation assay based on nucleic acid sequence based amplification (NASBA), for samples collected until 2001 and by the branched-chain DNA assay (Versant HIV RNA test, Version 3.0, lower limit of quantification 50 copies/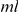; Bayer Diagnostics, Milan, Italy) from 2001 until 2008.
